# Impact of Geriatric Emergency Fellowship Training on the careers of Emergency Physicians

**DOI:** 10.7759/cureus.17903

**Published:** 2021-09-12

**Authors:** Phraewa Thatphet, Tony Rosen, Fae Kayarian, Lauren Southerland, Colleen M McQuown, Scott Dresden, Shan W Liu

**Affiliations:** 1 Emergency Medicine, Massachusetts General Hospital, Boston, USA; 2 Emergency Medicine, Khon Kaen University, Khon Kaen, THA; 3 Emergency Medicine, Weill Cornell Medical College, New York, USA; 4 Emergency Medicine, The Ohio State University Wexner Medical Center, Columbus, USA; 5 Department of Emergency Medicine, Louis Stokes Cleveland VA Medical Center, Cleveland, USA; 6 Emergency Medicine, Northwestern University Feinberg School of Medicine, Chicago, USA

**Keywords:** geriatric emergency, geriatric emergency medicine, geriatric emergency fellowship, fellowship training, career impact

## Abstract

Introduction

The geriatric population continues to increase and will impact the emergency department (ED). Older adult patients require different care from other groups of patients. Hence, it is essential to create a workforce that specializes in geriatric emergency medicine (GEM). Geriatric emergency medicine fellowships were developed to serve this need. However, despite 20 years since the creation of GEM fellowships, it is not known how GEM fellowships have impacted the career of graduates of GEM fellowships. The goal of this study is to examine the impact of these geriatric emergency fellowship training programs on the career of geriatric emergency fellows.

Methods

We surveyed the emergency physicians who had graduated from GEM fellowship programs in the US and Canada by using a 36-question, web-based questionnaire. The survey was pilot-tested on five GEM experts, fellowship graduates, and a GEM fellowship director.

Result

We had a 68% survey completion rate, two partially answered the study. All participants reported that they continue to have GEM as a part of his/her career. More than half either received grants, published papers, helped establish GEM divisions or caring in their hospital, and worked beyond clinical work in the ED, including academic and administrative fields. More than 80% reported that their fellowship helped obtain their current positions and was helpful in career progression. Approximately two-thirds were satisfied with their current work/life balance.

Conclusion

The GEM fellowship training has been impactful in the careers of former GEM fellows and has contributed to many becoming leaders in GEM clinical service, administration, education, and research. It can serve as a stepping stone to a leadership position in a GEM career. Furthermore, our study demonstrates that GEM graduates report high levels of career and clinical satisfaction.

## Introduction

The geriatric population continues to grow in magnitude as the average life expectancy increases. By 2050, it is estimated that the older adult population (aged ≥65) in the US will reach 83 million, and the oldest cohort of that group (aged ≥85) will triple in size compared to 2014 [1]. The growing geriatric population will impact the emergency department (ED) setting as the percentage of older patients presenting to the ED is estimated to increase from 20% to 33% in 2030 [2,3]. Older adult patients require different medical and psychosocial needs compared to younger patients as their physiology changes, and have difficulty in communication, increasingly take multiple medications, and develop geriatric syndromes such as dementia, frailty, and falls [2,4]. These differences lead to increased utilization of ED resources, laboratory studies, imaging tests, and social services [2,5]. It is increasingly important to create a workforce that specializes in geriatric emergency medicine (GEM). Unfortunately, emergency physicians (EPs) have not historically received adequate training to care for older adult patients [6,7].

Therefore, there is a great need to train a sub-group of ED physicians to lead improvement initiatives that promote the quality of care for geriatric ED patients. The first GEM fellowships were developed approximately 20 years ago to serve this demand. Over the past two decades, several academic institutions have offered these fellowships in the US and Canada. These opportunities present as clinical fellowships, which have intense clinical rotations in geriatrics and emergency medicine, and as non-clinical research fellowships. The Society of Academic Emergency Medicine (SAEM) promotes standardization of non-Accreditation Council for Graduate Medical Education (ACGME) approved postgraduate training opportunities for emergency medicine residency graduates, and eligible programs can apply for SAEM endorsement [8]. Currently, there are a total of seven GEM fellowship training programs active in the US and Canada. Five programs are post-residency fellowships, and three of these programs offer an option of a two-year fellowship with a fully-funded master’s degree [9]. There have been several studies of other emergency medicine fellowship experiences and their impact on their post-fellowship career [10,11].

However, despite 20 years since the creation of GEM fellowships, it is not known how GEM fellowships have impacted the career of graduates of GEM fellowships. We sought to examine the impact of these GEM fellowship training programs on careers by surveying GEM fellowship graduates. We also sought to understand how SAEM’s Academy of Geriatric Emergency Medicine (AGEM) could support GEM fellowships.

This article was previously presented as a meeting abstract poster at:

The MGH Clinical Research Day 2020 on October 1, 2020

The New England Regional meeting of SAEM (NERDS21) on April 7, 2021

The Society of Academic Emergency Medicine (SAEM) 2021 on May 12, 2021

## Materials and methods

Study design and population

To assess the impact of GEM fellowships, we surveyed the EPs who had graduated from GEM fellowship programs. We contacted all current and previous GEM fellowship directors of the seven active and one inactive programs in the United States and Canada that were listed in the study by Rosen et al. [9], for the contact information of GEM fellowship graduates from their programs. This study was reviewed and approved by our hospital institutional review board (approval #2019P001856).

Survey development

The questions were developed by the authors who have expertise in survey question design corresponding to the surveying emergency medicine guideline [12]. We reviewed and adapted the survey from previous studies evaluating fellowship graduates’ current work, responsibility in the ED and beyond, grants and publications, satisfaction in post-training career path, and work-life balance [13-16]. Our final survey had 36 questions (see appendix A) to assess the GEM fellows’ experience within the fellowship and their careers since graduating. We also included an open-ended question for suggestions they had for future GEM fellowships and how AGEM can support fellowships or residents considering fellowships. The survey queried participants on their post-training academic productivity, career path, their contributions to GEM, as well as their opinion on the quality of their GEM fellowship training. Survey data were collected and managed using a web-based software platform, REDCap (Research Electronic Data Capture), an electronic data capture tool (Vanderbilt University, Nashville, Tennessee, USA) hosted at Massachusetts General Hospital [17]. We pilot tested our survey on five GEM experts, including fellowship graduates and GEM fellowship directors and subsequently edited it to improve clarity. After the pilot testing, we re-arranged the sequence of questions and made the questions clearer without adding or deleting any item.

Survey administration

An email and link to a REDCap survey were sent out a total of three times, each one week apart. The fellows who did not have a current contact email or were non-responsive were excluded from the study.

Data analysis

We analyzed the results of the participants’ post-training academic productivity, career path, their GEM contribution, as well as their opinions regarding the quality of their GEM fellowship training. All results were analyzed in a blinded fashion. We used descriptive statistics. Percentages and means were used to describe normally distributed data; otherwise, we used medians and interquartile ranges (IQR).

## Results

We identified 25 overall geriatric emergency fellow graduates from eight institutions. We had a 68% (17/25) completion rate. A total of 19 participants (76%, 19/25) responded to the survey but two did not complete all answers. Six graduates did not respond, even though we tried to contact them and sent reminder emails a total of three times. All participants consented to participate in the study. Table 1 shows the demographic characteristics of the fellowship graduates and information about their GEM fellowship training. Those who completed the survey (N=17) had a mean age of 40.7 years old. Both SAEM-approved and non-SAEM fellowship programs had a similar number of graduates. Seven responders completed their fellowship five to 10 years ago, which is the majority group followed by those who graduated two to five years (N=6), one to two years (N=2), and less than one year ago (N=2). Nearly all GEM fellows had a clinical focus (88%, 15/17), and only a few (12%, 2/17) received a master’s degree as a part of the fellowship. The duration of the fellowship program varied from six months to two years.

**Table 1:** Demographic data and training SAEM=Society of Academic Emergency Medicine

	N(%) (N=17)
Mean age in years (SD)	40.7 (8)
Time after having graduated (in years)	
<1	2 (11.8)
1-2	2 (11.8)
2-5	6 (35.3)
5-10	7 (41.2)
Type of geriatric fellowship	
SAEM-approved fellowship	7 (41.2)
Non-SAEM fellowship	8 (47.1)
Not sure	2 (11.8)
Clinical fellowship	15 (88.2)
Training period	
Less than one year	1 (5.9)
One year	11 (64.7)
Two years	5 (29.4)
Did fellowship at the same institution as residency training	7 (41.2)
Received a master’s degree as part of the fellowship	2 (11.8)

Table 2 displays the current work status of participants. The GEM fellows maintained a position in GEM as a component of their careers. The estimated clinical hours of these past GEM fellows are 22.4 hours per week. There are various roles of graduated GEM fellows, including clinical, administrative, research, teaching, and service fields. Almost all of the graduated fellows taught GEM content in the past three years. About three-fourth (77%, 13/17) of the responders work in the institutions that are seeking, received, or interested in Geriatric Emergency Department Accreditation (GEDA), most (77%, 10/13) of the fellows are working in the roles of a champion, director, leader, or chair of the geriatric ED. More than half of the participants received grants and published papers following their GEM fellowship. 

**Table 2:** Current reported work EM=Emergency Medicine, ED=Emergency Department

	N (%) (N=17)
Continue to have geriatric emergency medicine as a part of their career	17 (100)
Work involved	
Clinical	16 (94.1)
Academic	13 (76.5)
Teaching	13 (76.5)
Administration	10 (58.8)
Current position	
Academic EM	13 (76.5)
Community EM	2 (11.8)
Other	2 (11.8)
Academic rank	
No academic rank	2 (11.8)
Clinical Instructor/Clinical Professor	4 (23.5)
Assistant Professor	9 (52.9)
Associate Professor	2 (11.8)
Currently in the academic track	11 (64.7)
Clinical educator track	3 (27.2)
Research track	3 (27.2)
Clinical track	1 (9.1)
Administrative track	1 (9.1)
Other	2 (18.2)
Currently in the tenured track	
Yes	1 (9.1)
Tenured track but not yet tenured	2 (18.2)
No	8 (72.7)
Current distribution effort	%Average
Clinical	53.8
Administrative	24.6
Research	21.4
Teaching	13.2
Service	5.2
Estimated clinical hours per week (SD)	22.4 (10)
Current institution seeking/received/interested in Geriatric Emergency Department Accreditation	13 (76.5)
Current champion/director/leader/chair of the geriatric ED	10 (76.9)
Taught geriatric EM content in the past three years	16 (94.1)
Received grant(s)	10 (58.8)
Received grant(s) after finishing fellowship training	7 (70)
Number of published papers (range)	8.6 (0-45)
Number of published papers after finishing fellowship training (range)	5.4 (0-27)

The participants’ work beyond their institutional level is shown in Table 3. More than half of the graduated fellows produced geriatric educational material for distribution beyond institutions and engaged in peer-reviewed medical journals. Some participants served at the hospital, state, regional, or national committee level. Participants who attended geriatric or EM conferences/educational events presented geriatric-specific content at those events. 

**Table 3:** Work beyond emergency department level EM=Emergency Medicine, ACEP= American College of Emergency Physicians, SAEM=Society of Academic Emergency Medicine, ED=Emergency Department

	N (%) (N=17)
Produced geriatric educational material for distribution beyond institutions	9 (52.9)
Currently engaged in	
Reviewer for a peer-reviewed journal in EM	9 (52.9)
Reviewer for a peer-reviewed journal in geriatrics	5 (29.4)
Decision editor or editorial board member for a peer-reviewed journal in EM	1 (5.9)
Member of local Institutional Review Board	1 (5.9)
Serve/have served national committees (e.g. ACEP, SAEM) in the past three years	14 (82.4)
Leadership position	7 (50)
Serve/have served state/regional committees (e.g. state ACEP, regional SAEM) in the past three years	4 (23.5)
Leadership position	1 (25)
Serve/have served hospital committees in the past three years	12 (70.6)
Leadership position	5 (41.7)
Geriatric activities participated in institution level in the past three years	
Participating in quality improvement	11 (64.7)
Writing protocols	11 (64.7)
Applying/Applied for Geriatric ED Accreditation	9 (52.9)
Running a formal educational course	7 (41.2)
Non-EM geriatric medical programs (e.g. Fall prevention clinic, geriatric consult)	5 (29.4)
Sit on geriatric-specific committees	5 (29.4)
Lead committee	3 (60)
Current institution has a GEM fellowship	6 (35.3)
Involved as a/an	
Mentor	3 (50)
Director	2 (33.3)
Assistant director	1 (16.7)
Lecturer	1 (16.7)
Current institution doesn’t have a GEM fellowship but is considering starting a GEM fellowship	6 (54.5)
Attended geriatric or EM conference/educational events since fellowship	13 (76.5)
Present geriatric-specific content at the meeting	9 (69.2)
Didactic	8 (88.9)
Abstract	7 (77.8)
Innovation	3 (33.3)
Other: Panelist, business meeting presentations, pre-conference workshops	1 (11.1)

Table 4 describes the opinions of the participants who completed GEM fellowships. The main factor in choosing a GEM fellowship for participants was the ability to serve the geriatric patient population, followed by mentorship, future job opportunities, work/life balance, research opportunities, and administrative opportunities. Most (82%, 14/17) thought their GEM fellowship helped them obtain their current positions, and 88.2% thought their GEM fellowship was very helpful for their career progression. Almost all (94%, 16/17) responded as “very satisfied” or “satisfied” with career progression after completing a fellowship, and 64.7% responded as “very satisfied” or “satisfied” with current work/life balance. Three-fourths of the participants planned to stay in the geriatric field for the next five years. 

**Table 4:** Opinion on geriatric emergency medicine

	N (%) (N=17)
Think fellowship helped obtain current positions	14 (82.4)
Think geriatric fellowship was very helpful or helpful for career progression	15 (88.2)
Very satisfied or satisfied with career progression after completing a fellowship	16 (94.1)
Very satisfied or satisfied with current work/life balance	11 (64.7)
Value received from fellowship	
Mentorship	14 (82.4)
Research opportunities	13 (76.5)
Administrative opportunities	11 (64.7)
Conference/educational opportunities	11 (64.7)
Will probably be in the geriatrics field for the next five years	13 (76.5)

Table 5 shows selected comments about geriatric fellowship training and how AGEM can support fellowships or residents considering fellowships. We did not include redundant comments. Graduated GEM fellows shared how they benefitted from the work they completed during their GEM fellowship and aided their career path. They also noted that their training made older adult patients they treated during their fellowship feel more comfortable with the quality of care they were receiving. Participants mentioned additional funding opportunities and promotion of the GEM fellowship program to EM residents might enhance future interest in applying for these programs. The graduated GEM fellows voiced their concerns about finding a suitable workplace environment after completing a GEM fellowship. For the full comments list, please see appendix B.

**Table 5:** Comments about geriatric fellowship training GEM=Geriatric emergency medicine, EM=Emergency medicine, ED=Emergency department, AGEM= Academy of Geriatric Emergency Medicine, SAEM= Society of Academic Emergency Medicine

Comments about geriatric fellowship training
Completely aligned with the demographics and their problems in the healthcare system, very innovative, and the potential for positive impact with small changes is very high.
I have had the great opportunity to learn about GEM research. The only concern is about the implementation of GEM for a resource-limited place.
My older patients love that I completed this fellowship - it comforts them to know that there is specialized interest in the health and wellness of older adults. Despite the fact that I did not make as much professional use of my training and research as I should have, the personal returns on my investment are immeasurable.
The GEM fellowship was more beneficial to my career than I had originally thought. Applicable to the community as well as academia.
I think that the geriatric EM fellowship was transformative for my career. It highlighted the issues and challenges in the care currently provided to older adults in the ED and broadened my experience and perspective on how to optimize geriatric care. It served as an opportunity for me to develop my research program both with the training, expectations and protected time. I was able to attend national conferences and meet, collaborate, and learn from national leaders. Through my fellowship, I was also able to collaborate with and join a team of more senior ED physicians who were interested in geriatric EM and who had previously completed a GEM fellowship. I have stayed on as faculty at the institution at which I trained and these colleagues have continued to be colleagues, collaborators, and close friends. Their mentorship, collaboration, and support have been critical for my career.
Benefits: Specialized education in a growing field, improved relationships/networking with geriatrics within the hospital system, able to integrate knowledge into ED processes, and resident education. Concerns: Hospital/administration buy-in, does require additional resources to support a geriatric ED.
How AGEM can support fellowships or residents considering fellowships
Provide funding opportunities or a data bank of funding opportunities
Give at least one GEM curriculum hour for EM residents (Formal) and a test for EM residents. They may be aware of GEM.
I think further outreach to EM residents to explain the benefits of a GEM Fellowship would be helpful for increasing the number of interested applicants.
Better publicity of the application process for GEM fellowships and advertising unfilled opportunities
Finances are usually a key concern of applicants
Continue to promote the potential for fellowship training to develop the next generation of academic and clinical leaders in geriatric EM. Support institutions that are considering developing geriatric EM fellowships, including fellowships with a specific focus, such as administration. Ensuring that existing fellowships are supported and maintained, with a goal that each training program has fellows each year. Ensure that interested residents and students are supported and encouraged to choose geriatric EM fellowship training.
Continuing to support a scholarship for residents and now fellows to SAEM. Advertising and highlighting the different fellowships and their offerings, focusing on the benefits of completing a fellowship and what it can do for you/your career.

## Discussion

Our research shows that GEM fellowship training has been impactful in the careers of former GEM fellows and has contributed to many becoming leaders in GEM clinical service, administration, education, and research. Our study is the first to show the value of pursuing GEM fellowship training. These fellowships vary in type, duration, and focus [9], which allows for a customizable experience.

Geriatric emergency medicine is a growing field and needs more clinicians with specialized training to lead as administrators, educators and, researchers [2,18]. With the advent and growth of GEDA [19], many EDs will have to require a GEM physician champion/leader. Former GEM fellows are ideally positioned to serve in this role, and the results of this study suggest that many are already doing so. Three-quarters of the graduated fellows have become medical directors for their hospital’s geriatric ED. While clinical practice is the main component of their work, most GEM fellowship graduates also report devoting time to teaching, research, and administration, suggesting that fellowships are training physicians for careers integrating multiple roles. Graduated fellows still incorporate GEM as a part of their career, suggesting that in-depth exposure to GEM during their training highlighted its importance and supported their interest in pursuing it. That many of the graduated fellows have had papers published in peer-reviewed journals and received grants suggest that GEM fellowships have also begun to develop a specialized workforce that may advance the field with important new practice-changing discoveries. This finding is similar to previous studies that revealed that completing a subspecialty fellowship is associated with an increase in academic paper publications and grants funding [20-22]. Comments from former fellows about their training further emphasize the impact it has had on their careers - “The GEM fellowship was more beneficial to my career than I had originally thought. Applicable to the community as well as academia.” And “I think that the geriatric EM fellowship was transformative for my career. It served as an opportunity for me to develop my research program both with the training, expectations, and protected time.”

Our study suggests that GEM fellowship graduates not only continue to work in GEM, but also have an impact on GEM beyond their institution. For instance, graduated GEM fellows have gone on to present content on GEM at national conferences, serve on national/regional/state committees, and serve as reviewers for peer-reviewed journals in the emergency and geriatric fields. 

Our work underscores the value of post-residency fellowship training in GEM. Fellows develop a niche area of expertise, mentorship opportunities, unique qualifications for future jobs, and experience with research and administrative work. Karpinski et al. [13] measured the various reasons individuals seek fellowship training, which prominently included academic productivity, clinical expertise, recruitment and reputation, clinical productivity, and the scholarly environment. Clearly, current, existing GEM fellowships serve this role, as most fellows believe that their fellowship training helped obtain their current positions and career progression. 

Approximately 64.7% of the GEM fellows reported being “very satisfied” or “satisfied” with their current work-life balance, which is much higher than overall emergency physicians who have the satisfaction rate at about 50% [23]. They also reported working an average of 22.4 clinical hours per week, which is less than the majority of emergency physicians. GEM fellowship training may lead to a more balanced career with a smaller likelihood for burnout since research shows that too much workload and less recovery time from work are associated with burning out [23-27]. Also, Shanafelt et al. and Geurts et al. reported that satisfaction of work-life balance significantly impacts physician burnout [23,28]. Overall, about three-fourths of the graduated fellows think they will still be working in the geriatrics field for at least the next five years.

While most of the GEM fellows reported multiple beneficial aspects of their training, issues were raised about choosing a GEM fellowship that merits further exploration. One participant mentioned that working in a hospital with limited resources and academic opportunities since graduating from his GEM fellowship has been frustrating. He recommended that potential applicants ensure that the GEM fellowship training is an appropriate fit for their future career or hospital setting before committing. Finding the faculty position that supports GEM improvements will maximize the efficacy of fellows after graduating from GEM fellowship programs. Many hospitals are seeking the Geriatric Emergency Department Accreditation (GEDA) program [29] to improve the ED care of geriatric patients. These hospitals may particularly recruit GEM fellowship-trained physicians. To ensure the highest quality care provided to its geriatric emergency patient populations, the hospital may have to provide additional resources both for academic and healthcare service purposes. Such as an ED that has a geriatric-friendly environment, tools for screening specific risks for older adults, and healthcare workers who devote themselves to geriatric patients. 

As AGEM is focused on supporting GEM fellowships, the survey solicited ideas on how this support might be best operationalized. Suggestions such as increasing exposure to GEM by adding it to the EM residency curriculum should be incorporated into AGEM’s strategic plan. An article by Ringer et al. recommended increasing the proportion of GEM content in the EM board certification and licensing examinations [30]. AGEM also recently created a scholarship for fellows to encourage GEM research. Future AGEM projects should encourage medical students/residents to pursue a specialization in GEM and thus support GEM fellowships. 

Limitations

Our study has several limitations. First, the sample size is relatively low, compared to other fellowship studies [13-15]. While GEM fellowships have existed for about 20 years, the first 10 years had only one institution with a GEM fellowship. Furthermore, three of the authors served as pilot testers and research participants. We only received responses from 76% of fellowship graduates. It is possible that non-respondents felt very differently about their GEM fellowship training, which may have influenced their decision not to respond, biasing our results. We had intended initially to report quantitatively on grants received and published articles by fellows, but several respondents answered “numerous” or “many” rather than an exact number, precluding this. Further, since we blinded responses, we could not reach out to respondents to clarify their answers. Among the 19 respondents, two did not complete the entire survey. We analyzed and compared the results between the participants who had completed the study with the included incomplete surveys and noted that it did not have a significant difference in the results. Additionally, in a survey about the current career and the impact of an elective training program, social desirability response bias may have impacted responses regarding “work-life balance” and the “success after training”. We believe, though, that the narrative comments provided by respondents reinforce the impact of this training and their current careers.

## Conclusions

Our research supports that GEM fellowship training programs can serve as a stepping stone to a leadership position in a GEM career. Furthermore, our study demonstrates that GEM graduates report high levels of career and clinical satisfaction. Geriatric emergency medicine fellowship training programs have a positive impact on the career of GEM fellowship graduates and considering the growth in geriatric EDs and emergency medicine, residents should strongly consider this post-residency option as a future career path.

## Appendices

Appendix A

Geriatric Fellowship Survey

[] I agree to participate in this research

1.     Fellowship Name (optional)

        Age_____

2. Describe your geriatric fellowship

            1.  SAEM approved

            2. Non-SAEM EM fellowship

            3. Non-EM fellowship

2.     How long was your fellowship period?

           1. Less than one year

           2. One year

           3. Two years

           4. Three + years

3. How long ago did you finish your fellowship training?

             1. <1 year ago

             2. One to two years ago

             3. Two to five years ago

             4. Five to 10 years ago

4. Was your fellowship at the same institution as your residency training?

            Yes/No

5. Did you receive a master's degree as part of your fellowship?

            Yes/No

6. Is geriatric emergency medicine still part of your career?

            1. Yes, I am involved in  (check all that apply)

            a. Academic

            b. Clinical

            c. Teaching

            d. Admin 

            2. No. I am

            a. Nonclinical

            b. Practice limited to patients <60 years old

            c. Do not practice medicine

            d. Practice non-EM medicine

7. Current position

           1. Academic EM

           2. Community EM

           3. Other 

       Please specify____________

8. Do you think your fellowship helped you obtain your current positions?

            Yes/No

9. Academic rank

            1. Clinical Instructor/Clinical Professor

            2. Assistant Professor

            3. Associate Professor

            4. Professor

            5. No academic rank

10. Title (e.g. Director of Research, Geriatric EM Director)

            Open text with character limit

Are you currently in an academic track?

            Yes/No

11.  Academic Track

            1. Research track

            2. Clinical educator track

            3. Clinical track

            4. Administrative track

            5. Other, specify __________

12. If on an academic track, are you currently

            1. Tenured

            2. Tenured track but not yet tenured

            3. Non-tenure track

13. Current distribution effort (by %, must =100)

            1. % Research

            2. % Administrative

            3. % Teaching

            4. % Service (e.g. National committees)

            5. % Clinical

            6. % Other, please specify_______________

14. Estimated clinical hours per week on average ______

15. Is your institution seeking/received/interested in Geriatric Emergency Department Accreditation?

            Yes/No

            A. If yes, are you the champion/director/leader/chair of the Geriatric ED?

            B. If no, is this something that you are planning on proposing to hospital leadership?

16. In the past three years have you taught Geriatric EM content formally to (check all that apply)

            1. EM residents

            2. Non-EM residents

            3. Medical students

            4. Attending EM faculty

            5. Non-EM attending faculty

            6. Prehospital providers

            7. Nurses

            8. Medical interdisciplinary 

            9. Community members

            10. Industry

17. In the past three years have you produced geriatric educational material for distribution beyond your institutions? Check all that apply

            1. Video didactics

            2. Podcasts

            3. Non-PubMed publications

            4. YouTube video

            5. Other,  please specify________

18.  Do you currently engage in any of these academic leadership activities? Check all that apply

            1. Decision editor or editorial board member for peer-reviewed journal in EM

            2. Decision editor or editorial board member for peer-reviewed journal in geriatrics

            3. Decision editor or editorial board member for peer-reviewed non-EM, non-geriatrics journal

            4. Reviewer for peer-reviewed journal in EM

            5. Reviewer for peer-reviewed journal in geriatrics

            6. Reviewer for peer-reviewed journal non-EM, non-geriatrics

            7. Grant reviewer for foundation

            8. NIH/AHRQ (National Institutes of Health/Agency for Health Research and Quality) study section member

            9. Member of local Institutional Review Board

19. Number of national committees (e.g. ACEP, SAEM) on which you serve/have served in the past three years

            ____________         

      Number of national committees in leadership position

            ____________

20. Number of state/regional committees (e.g. state ACEP, regional SAEM) on which you serve/have served in the past three years

_________________________

21.Number of hospital committees on which you serve/have served in the past three years

_________________________

22. At an institution level, have you participated in any of the following geriatric specific activities in the past three years. Check all that apply

            1. Running a formal educational course

                        1a. If yes, for whom? 

            2. Participating in quality improvement

            3. Writing protocols

            4. Applying/applied for Geriatric ED Accreditation

            5. Non-EM geriatric medical programs (e.g. Fall prevention clinic, geriatric consult)

            6. Sit on geriatric specific committees 

                        6a. If yes, specify the committee 

                        6b. If yes, did/do you lead the committee?

23. Does your current institution have a geriatric EM fellowship?

            Yes/No

            A. if yes, how are you involved? (May check more than one)

                        i. Director

                        ii. Assistant director

                        iii. Lecturer

                        iv. Mentor

            B. If no, are you considering starting a fellowship? Yes/No

24. Have you ever received any grants? Yes/no. Please list your grants received including organization, type of grant, duration__________________

25. How many grants did you receive after finishing fellowship training? _______

26. How many papers have you published? ____since finishing fellowship training

27. Have you attended any geriatric specific conferences/ meetings since your fellowship

            A. If yes, have you presented geriatric-specific content?

                        If yes, check type

                        a. Didactic

                        b. Abstract

                        c. Innovation

                        d. Other          

28. In your opinion, for your career progress, completing a geriatric fellowship was

            1. Very helpful

            2. Helpful

            3. Neutral

            4. Not helpful

29. How satisfied are you with your career progress since completing your fellowship?

            1. Very satisfied

            2. Satisfied

            3. Neutral

            4. Not satisfied 

            5. Very unsatisfied 

30.   Please describe your geriatric plans for the next five years

                        1. I will probably be in geriatrics 

                        2. I will probably not be in geriatrics

                        3. I don’t know

31. What value did you get from your fellowship? Check all that apply

            1. Mentorship

            2. Research opportunities 

            3. Administrative opportunities

            4. Conference/educational opportinutes

            5. Other, please specify

32. What were the biggest factors in choosing your fellowship institution? Please range from most (1) to less (6) important factor

            1. Mentorship

            2. Research opportunities

            3. Administrative opportunities  

            3. Structure

            4. Location

            5. Work/life balance

33. Describe your satisfaction of your current work/life balance

            1. Very satisfied

            2. Satisfied

            3. Neutral

            4. Not satisfied 

            5. Very unsatisfied 

34. Please share any other opinions you have on your geriatric fellowship (benefits/ concerns)

            ______________

35. Please share any ideas on how AGEM can support fellowships or residents considering fellowships 

__________________

Appendix B

Full Comments List

- I wish I had stronger mentoring from EM. My fellowship director left a couple of months after I started my fellowship and I needed more guidance. Also, finances were a concern so the decreased pay opportunities made an impact.  

- (The fellowship program) completely aligned with the demographics and their problems in the health care system, very innovative, and the potential for positive impact with small changes is very high.

- I have a great opportunity to learn about GEM research. The very concern is about the implementation of GEM for a resource-limited place.

- I went into my fellowship thinking that I would gain some knowledge about polypharmacy and transitions of care but thought I already knew a lot. I was so wrong and learned so much. I enjoy passing what I know on to our EM residents. 

- It was an amazing experience, and I am very happy to promulgate geriatric EM in all academic domains.

- The Toronto program is not SAEM-certified and ideally should be. The SAEM should strike a working group with the Royal College of Physicians of Canada to create a dual certification of the fellowships.

- Important: My fellowship was not in geriatrics, and my geriatrics training occurred as part of my GEMSSTAR, not my fellowship. So you may not want to include my responses.

- My older patients LOVE that I completed this fellowship - it comforts them to know that there is a value placed on my interest in the health and wellness of older adults. Despite the fact that I did not make as much professional use of my training and research as I should have, the personal returns on my investment are immeasurable.

- (The fellowship is) more beneficial to my career than I had thought, applicable to the community as well as academia. 

- I think that the geriatric EM fellowship was transformative for my career. It highlighted the issues and challenges in the care currently provided to older adults in the ED and broadened my experience and perspective on how to optimize geriatric care. It served as an opportunity for me to develop my research program both with the training, expectations, and protected time. I was able to attend national conferences and meet, collaborate, and learn from the national leaders. Also, I was able to, through my fellowship, collaborate with and join a team of more senior ED physicians who were interested in geriatric EM and who had previously completed a GEM fellowship. I have stayed on as faculty at the institution at which I trained and these colleagues have continued to be colleagues, collaborators, and close friends. Their mentorship, collaboration, and support have been critical for my career.

*EM=Emergency medicine, GEM=Geriatric emergency medicine, SAEM=The Society for Academic Emergency Medicine, ED=Emergency Department, GEMSSTAR=Grants for Early Medical/Surgical Specialists' Transition to Aging Research
